# Outcomes of the Treatment of Humeral Shaft Fractures by Closed Reduction and Internal Fixation With Multiple Intramedullary Kirschner Wires (K-wires)

**DOI:** 10.7759/cureus.51009

**Published:** 2023-12-23

**Authors:** Adham M Abdulsamad, Turki Al Mugren, Mohammed T Alzahrani, Faisal T Alanbar, Turki A Althunayan, Abdullah Mahayni, Abdulrahman H Alfarag, Mohammad T Alotaibi, Musab Almuqbil, Ahmed H Alfarraj

**Affiliations:** 1 Department of Surgery, Division of Orthopedic Surgery, King Abdulaziz Medical City, Riyadh, SAU; 2 Department of Orthopedic Surgery, King Saud Bin Abdulaziz University for Health Sciences College of Medicine, Riyadh, SAU; 3 Department of Medicine, King Saud Bin Abdulaziz University for Health Sciences College of Medicine, Riyadh, SAU

**Keywords:** complications, k-wires, surgical treatment, fracture, humerus shaft

## Abstract

Background

Humeral shaft fractures are common orthopedic injuries, and their treatment options vary based on fracture characteristics. One surgical method involves closed reduction and internal fixation (CRIF) with multiple intramedullary (IM) Kirschner wires (K-wires), which remains less explored, especially in adults. This study aims to investigate the outcomes of the treatment of humeral shaft fractures by closed reduction and internal fixation with multiple flexible intramedullary K-wires.

Materials and methods

We conducted a retrospective study at King Abdulaziz Medical City, Riyadh, Saudi Arabia, focusing on patients with traumatic humeral shaft fractures who underwent flexible intramedullary K-wire fixation. We analyzed nine patient records for demographic information, fracture location, type, mechanism of injury, intra-operative and post-operative factors, and complications.

Results

Fractures mostly affected the middle third of the humerus (55.6%) and were primarily transverse or oblique (77.8%). Motor vehicle accidents were the leading cause of injury (66.7%). Intra-operative time was 125 minutes on average, with minimal blood loss (78 mL). No participants required intra-operative blood transfusion. Complications following IM K-wire fixation were absent in all cases. Three patients had not yet undergone instrumental removal, and most reported mild or no pain during the final follow-up. All participants achieved a full range of motion for their elbows. All participants achieved complete radiological and clinical union (healing) of their fractures.

Conclusion

The use of multiple intramedullary K-wires for the treatment of humeral shaft fractures in this study demonstrated positive outcomes with low complication rates. This approach provides an effective option for managing these fractures, particularly in cases where surgical indications favor it.

## Introduction

Fractures of the humeral shaft are relatively common, representing approximately 3% of all orthopedic injuries with an incidence rate of 19 per 100,000 persons per year [1]. It follows a bimodal age distribution, most commonly affecting females in the seventh decade mainly due to falls and osteoporotic changes, followed by males in the third decade usually as a result of high-energy trauma, which can lead to a serious burden on society [2]. Humeral shaft fractures can be classified based on the location to the proximal, middle, or distal third or based on the pattern: spiral, transverse, or comminuted. Risk factors for humeral shaft injuries include playing high-impact sports, osteoporosis and increasing age, motor vehicle accidents, and previous fractures [3]. One of the most common complications is radial nerve palsy, which has an incidence rate of 11.8% after a humeral shaft injury, making it the most common peripheral nerve injury related to long bone fractures [4].

The treatment of humeral shaft fractures varies from conservative (casting, humerus brace, or arm sling) management to surgical fixation depending on multiple factors including fracture characteristics, patient factors, and surgeon preference. Furthermore, surgical fixation involves a wide variety of methods and techniques, including but not limited to the use of rigid intramedullary (IM) fixation, plate and screw fixation, Kirschner wire (K-wire) fixation, and flexible intramedullary nail (IMN) fixation [5].

The surgical indications for the management of humeral shaft fractures can be absolute such as open fractures, severe soft tissue injury or bone loss, vascular injury requiring repair, brachial plexus injury, ipsilateral forearm fracture (floating elbow), compartment syndrome, and peri-prosthetic humeral shaft fractures. The relative indications for surgical management of humeral shaft fractures are mainly bilateral humeral fractures, poly-trauma, pathological fractures, burns or soft tissue injuries that preclude bracing, distraction at the fracture site, short oblique or transverse fracture patterns, and intra-articular extension [6,7].

Humeral shaft fractures can be treated conservatively if they meet the proper criteria, but there are problems associated with this management technique [3]. Firstly, the patient will be managed with a cast or brace, which might not allow him to perform an immediate range of motion exercises for his elbow and shoulder, increasing the risk of patient discomfort and stiffness. Secondly, the non-surgical management of humeral shaft fractures has a risk of non-union or malunion. Many of these fractures might not heal after a period of immobilization and will need surgical fixation, which causes more distress and dissatisfaction for the patient. Lastly, patient cooperation is very important for the success of this treatment method, where an uncooperative patient can increase the risk of failure of management [6].

With regard to the surgical fixation of humeral shaft fractures, open reduction and internal fixation (ORIF) with plates and screws has its own set of problems and possible complications. A larger wound size is needed to properly expose the fracture and fix it, which may increase the risk of wound complications and post-operative radial nerve palsy. Even with ORIF, there is still a risk for non-union or malunion due to the disturbance of the fracture biology and hematoma. Lastly, some patients have skin conditions (e.g., burns) that might increase the risk of wound complications with ORIF [8]. Surgical management with rigid antegrade intramedullary nailing carries the risk of impaired shoulder function due to the violation of the rotator cuff [9].

Our technique of flexible intramedullary K-wire fixation for humeral shaft fractures aims to provide the best possible functional and clinical outcome to patients with the least possible complications. Our technique allows the patient to start an immediate range of motion, which would theoretically decrease the risk of post-operative stiffness and patient discomfort. Furthermore, this technique uses a small and limited incision distally away from the fracture site, which theoretically decreases the risk of wound complications and decreases the risk for non-union as it preserves the fracture biology. Furthermore, this surgical technique does not require exposure and dissection of the radial nerve, which could decrease the risk of radial nerve palsy associated with ORIF. Our study and surgical technique are performed by a single surgeon in a single center in Saudi Arabia.

The use of flexible intramedullary K-wires in the surgical treatment of humeral shaft fractures is very limited in clinical practice, especially in the adult population. Furthermore, the literature is scarce on this topic, and there are only a handful of studies that address this surgical technique. There is a strong need for high-quality research that evaluates the outcomes of intramedullary K-wires. The aim of this research study is to study the outcomes of the treatment of humeral shaft fractures by closed reduction and internal fixation (CRIF) with multiple flexible intramedullary K-wires.

## Materials and methods

This research analyzed the medical records of 17 patients who received IM K-wire fixation for mid-shaft humeral fractures between 2017 and 2022. Eight patients were excluded from the study as their data was incomplete in their medical records (incomplete inpatient or outpatient documentation with regard to the variables needed for data collection), which left us with a sample size of nine patients. All patients included in this study were over the age of 14 and were admitted to the hospital due to a traumatic humeral shaft fracture. Our assessment focused on potential complications such as infection, wound dehiscence, stiffness, non-union, and neurovascular injury, as well as clinical and radiological union rates, the duration of hospital stay, time to union, surgical time for instrumentation removal, and intra-operative time. We obtained relevant patient data from the medical records system (BESTCare, Saudi Korean Health Informatics Company, Riyadh, Saudi Arabia) after receiving Institutional Review Board (IRB) approval. It is important to note that patient data was collected retrospectively without any intervention.

Study area

The study was conducted at the King Abdulaziz Medical City, Riyadh, Saudi Arabia.

Inclusion criteria

The inclusion criteria were patients above the age of 14 years old, patients admitted with humeral shaft fracture due to traumatic injury, and patients who underwent operative fixation of humeral shaft fracture by IM K-wires.

Exclusion criteria

The exclusion criteria were patients below the age of 14 years old, patients with humeral shaft fracture due to nontraumatic injury (e.g., pathological fracture), patients with humeral shaft fracture that was treated conservatively, patients with humeral shaft fracture that was treated operatively with fixation other than IM K-wires (e.g., plate and screws and external fixation), and patients with distal intra-articular humeral fractures.

Study design

This was a descriptive cross-sectional study evaluating patients presenting with humeral shaft fractures due to traumatic injury treated with flexible IM K-wires.

Sampling technique

Non-probability (nonrandom) sampling technique was used to include all patients with mid-shaft humeral fractures who were treated by flexible IM nail fixation.

Data collection methods, instruments used, and measurements

After obtaining approval from the Institutional Review Board (IRB), we reached out to the BESTCare medical record system to gather specific medical records of patients who met the study's inclusion and exclusion criteria. We utilized a comprehensive data collection form to gather key demographic information including age, gender, body mass index (BMI), comorbidities, and smoking habits, as well as fracture location. To pinpoint the fracture location, we divided the diaphysis (shaft) into three equal segments: the proximal segment near but not involving the shoulder, the mid-shaft segment in the middle of the humeral shaft, and the distal segment near but not involving the elbow. Additionally, we captured data on fracture type (simple, transverse, spiral, segmental, and comminuted), open fracture, the duration of surgery, time to union, the failure of fixation, prominent hardware post-operatively, blood transfusion intra-operatively, blood transfusion post-operatively, the range of motion post-operatively, non-union, infection post-operatively, the period of immobilization post-operatively, and time to removal. We also documented the date of fracture, surgery, and removal. Finally, we seamlessly transferred the data from the collection form to a predesigned Excel (Microsoft® Corp., Redmond, WA) sheet.

Data analysis

The necessary data was gathered into a data collection form from the BESTCare system and transferred from the data collection form to an already designed Excel sheet. A descriptive analysis was used to describe our objectives including the outcome and complications. Simple frequencies and percentages of the categorical variables were calculated and tabulated. For quantitative variables, means and standard deviations were illustrated. There were a total of nine cases. Eight patients were excluded from the study as their data was incomplete in their medical records.

Operative technique

**Figure 1:**
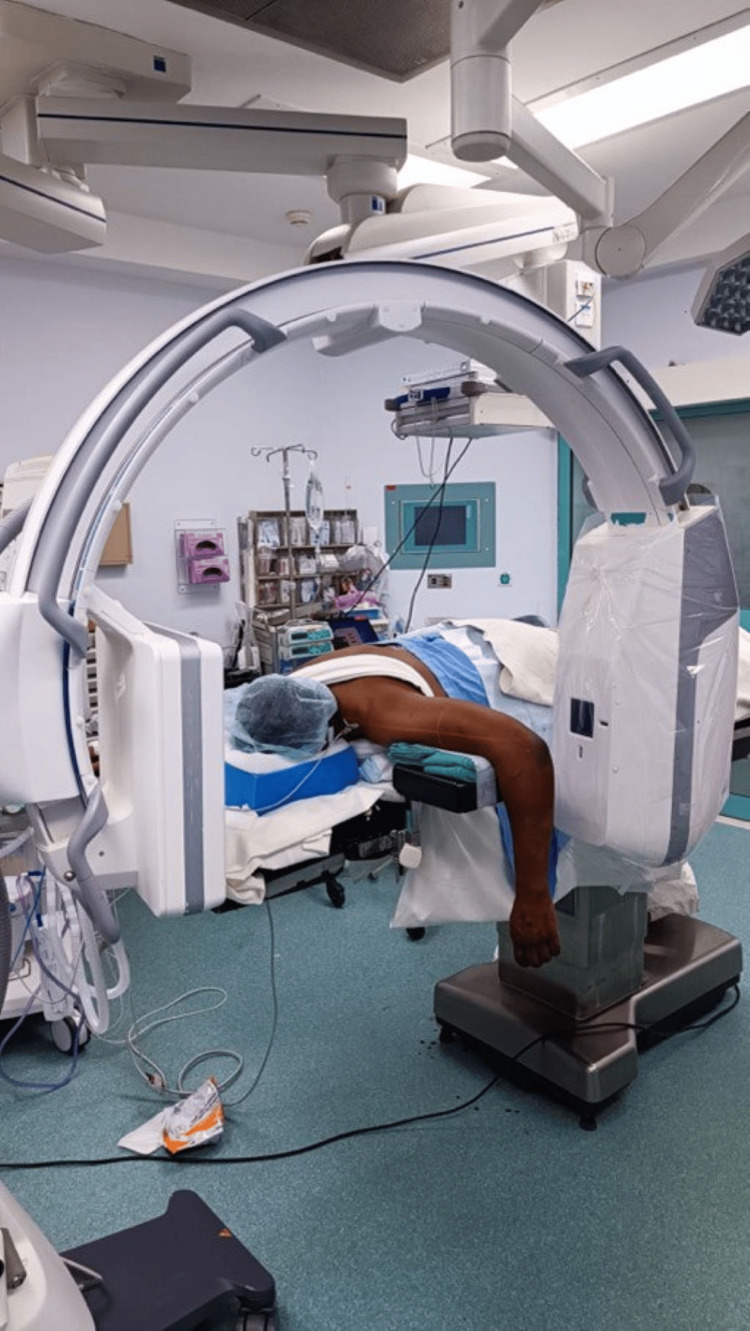
Prone positioning of the patient.

**Figure 2:**
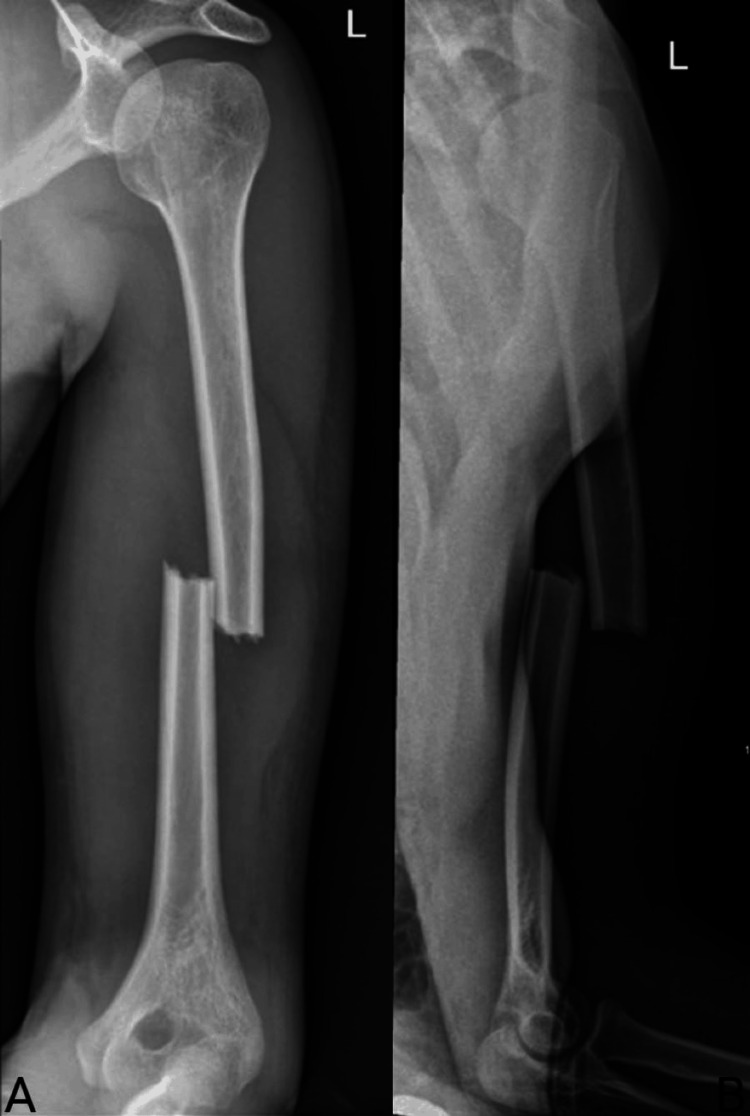
AP and lateral views of the left humeral shaft fracture. (A) AP view of the left displaced transverse humeral shaft fracture. (B) Lateral view of the left humeral shaft fracture. AP: antero-posterior

After inducing general anesthesia and intubating the patient, the patient is positioned in a prone position (some cases were done in a lateral decubitus position with the fractured side up) (Figure 1). X-rays are taken to ensure the proper visualization of the fracture site and the entire humerus (Figure 2). We then proceed to prepare and drape the surgical site in the usual sterile manner.

**Figure 3:**
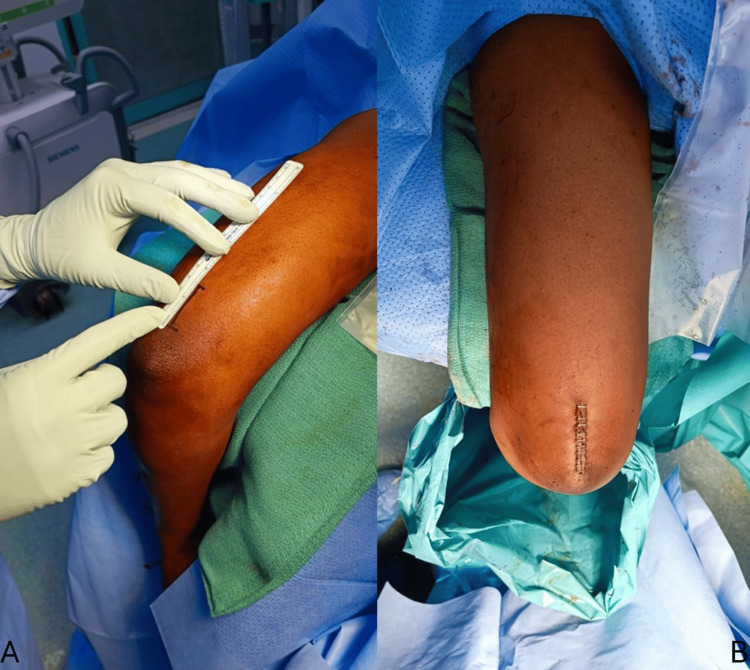
Intra-operative photographs showing the size of the wound utilized. (A) Measurement of the intended incision. (B) Incision site after procedure.

A posterior longitudinal incision (Figure 3), approximately 3 cm in length and just proximal to the olecranon fossa, is made. Dissection is performed down to the triceps tendon, and a longitudinal split is created through the triceps tendon. The dissection is then continued down to the humerus. A 1 × 1 cm bone window 2 cm proximal to the olecranon fossa is performed by drilling with a 4 mm drill bit in the posterior cortex; the widening of the window was performed using a bone awl to facilitate entry of the wires.

A flexible K-wire of 2-3.5 mm is bent at a three-point bending configuration (proximal bend, middle bend, and distal bend) in order to allow three-point stability in the intramedullary canal. This surgical technique involves flexible K-wires, not elastic nails.

**Figure 4:**
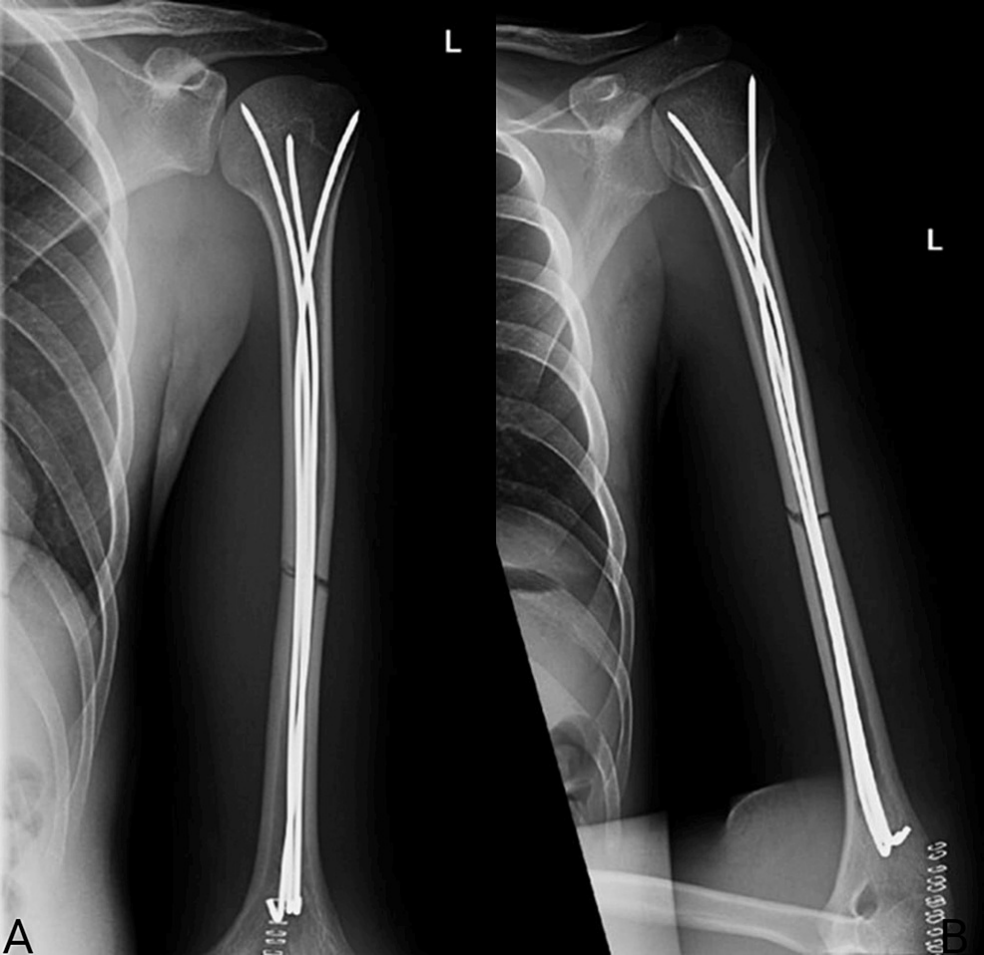
AP and lateral views of the left humeral shaft fracture post IM K-wire fixation with four K-wires. (A) AP view of the left humeral shaft fracture post IM K-wire fixation. (B) Lateral view of the left humeral shaft fracture post IM K-wire fixation. AP, antero-posterior; IM, intramedullary; K-wires, Kirschner wires

The wires are introduced into the bone window using a T-handle chuck and advanced past the intramedullary fracture under fluoroscopic guidance (antero-posterior {AP} and lateral views) while maintaining good reduction until the proximal humerus is reached. Two or three more K-wires are inserted in the same technique to fill at least 80% of the canal while spreading them proximally in a bouquet fashion (Figure 4). As many K-wires as needed are inserted to fill the canal. After confirming the proper alignment and rotation of the humerus, the distal ends of the K-wires are bent at 120° with a plier to prevent migration and not to be prominent. The wound is irrigated and closed layer by layer. The triceps tendon is closed with Vicryl 0 sutures, and the subcutaneous tissue is closed with Vicryl 2-0. The skin is closed with clips. A dressing is applied, and the patient is then repositioned to a supine position.

**Figure 5:**
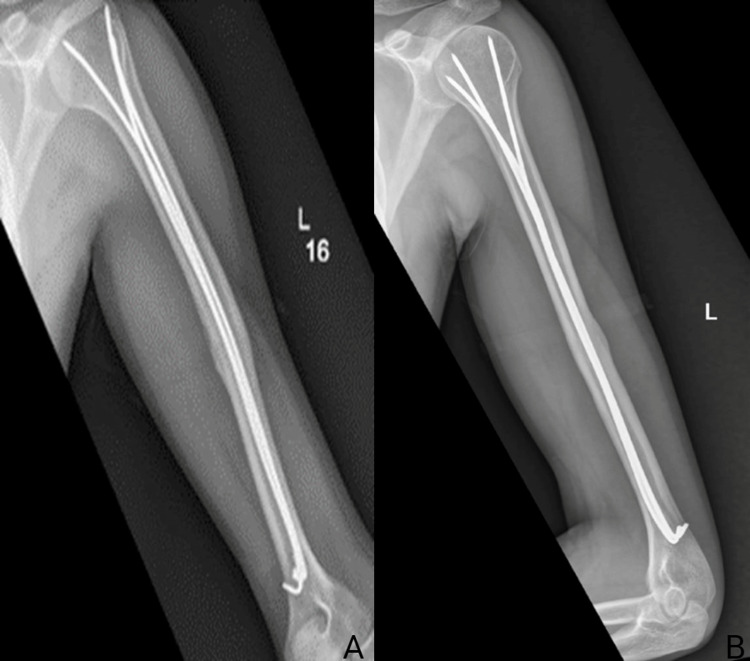
AP and lateral views of the left humeral fracture six months post fixation demonstrating complete healing of the fracture. (A) AP view of the left humerus. (B) Lateral view of the left humerus. AP: antero-posterior

**Figure 6:**
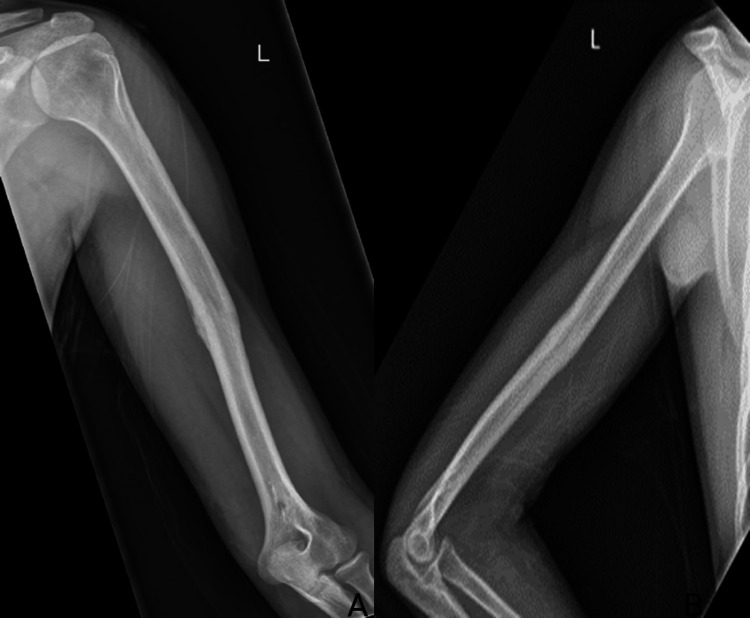
AP and lateral X-rays post removal of the K-wires. (A) AP view of the left humerus. (B) Lateral view of the left humerus. AP, antero-posterior; K-wires, Kirschner wires

Post-operatively, the patient may be given a functional brace just for his comfort and is allowed immediate passive range of motion exercises for the elbow and shoulder joints. The functional brace does not need to be given to all patients and does not add any stability. The patient is discharged the following day with an appointment scheduled for two weeks later for wound check and clip removal. After 2-3 weeks post-operatively, the patient is referred to physiotherapy for active shoulder and elbow range of motion. After the fracture is healed, strengthening is started. The patient is then followed up with regular outpatient visits involving clinical examination and X-rays (six weeks and then three months and then six months). Once the fracture has fully healed (Figure 5), the patient is scheduled for K-wire removal in the OR if the patient desires (usually after one year post fixation) (Figure 6). K-wire removal does not need to be done for every patient. If the patient is scheduled for removal, the procedure involves a simple short procedure in the OR under regional or general anesthesia.

## Results

Table 1 presents a comprehensive overview of the sociodemographic characteristics of the participants involved in the study, totaling nine individuals. The average age of the participants stands at 34 years, with a notable standard deviation of 20 years. The age range of the participants spans from 16 to 77 years. In terms of body mass index (BMI), the mean value is 30, with a standard deviation of 6. Gender distribution shows that most participants are male, constituting 88.9% of the sample, while one participant is a female (11.1%). Regarding comorbidities, three participants (33.3%) have diabetes mellitus, one (11.1%) has hypertension, and another one (11.1%) has dyslipidemia. Additionally, various other comorbidities such as Hodgkin lymphoma, ischemic heart disease, bronchial asthma, and deep venous thrombosis are present in four participants (44.4%), while four individuals (44.4%) do not exhibit any recorded comorbid conditions. The table also indicates that two participants (22.2%) are smokers.

**Table 1:** Sociodemographic characteristics of the participants in the study (n = 9). n, number; SD, standard deviation; BMI, body mass index

		n (%)
Age (mean ± SD)	34 ± 20	
BMI (mean ± SD)	30 ± 6	
Gender	Male	8 (88.9%)
	Female	1 (11.1%)
Comorbidities	Diabetes mellitus	3 (33.3%)
	Hypertension	1 (11.1%)
	Dyslipidemia	1 (11.1%)
	Others	4 (44.4%)
	None	4 (44.4%)
Smoking status	Smokers	2 (22.2%)

Table 2 provides a comprehensive overview of the clinical features observed in patients with humeral fractures in the study. The distribution of fractures across different segments of the humerus reveals that the middle third is the most commonly affected, accounting for 55.6% of cases, followed by the proximal third at 33.3% and the distal third at 11.1%. Notably, none of the participants displayed signs of radial nerve palsy prior to the operation. Furthermore, all fractures were classified as closed, with none being categorized as open fractures. In terms of fracture type, the majority were either transverse or oblique, constituting 77.8% of cases, while spiral fractures were observed in 22.2% of cases. Interestingly, there were no reported cases of comminuted fractures. The mechanisms of injury leading to these fractures varied, with motor vehicle accidents being the most prevalent cause, accounting for 66.7% of cases. Falls accounted for 22.2% of cases, while other miscellaneous causes (arm wrestling activity) constituted 11.1%.

**Table 2:** Clinical features of humeral fracture. n: number

		n (%)
Fracture location	Proximal third	3 (33.3%)
	Middle third	5 (55.6%)
	Distal third	1 (11.1%)
Radial nerve palsy pre-operatively	No	9 (100.0%)
Open fracture	No	9 (100.0%)
Fracture type	Transverse/oblique	7 (77.8%)
	Spiral	2 (22.2%)
	Comminuted	0 (0.0%)
Mechanism of injury	Fall	2 (22.2%)
	Motor vehicle accident	6 (66.7%)
	Others	1 (11.1%)

Table 3 outlines crucial intra-operative factors pertaining to the fixation of humeral shaft fractures using intramedullary (IM) K-wires. The mean duration of the intra-operative procedure was recorded at 125 minutes, with a relatively minor deviation as indicated by the standard deviation of 23 minutes. In terms of blood loss, the average amount was 78 mL, with a standard deviation of 51 mL. This demonstrates some variability in blood loss among the patients. Notably, none of the participants required a blood transfusion during the intra-operative period, indicating that the surgical procedures were successfully conducted without the need for additional blood support.

**Table 3:** Intra-operative factors related to IM K-wire fixation of humeral shaft fractures. n, number; SD, standard deviation; IM, intramedullary; K-wire, Kirschner wire

	n (%)
Intra-operative time during IM K-wire fixation (minutes) (mean ± SD)	125 ± 23
Blood loss (mL) (mean ± SD)	78 ± 51
Need for intra-operative blood transfusion	0 (0.0%)

Table 4 outlines significant post-operative factors in the context of intramedullary (IM) K-wire fixation for humeral shaft fractures. The first post-operative visit occurred at a mean of 20 days, exhibiting a standard deviation of 17 days. Instrumentation removal took an average of 13 months, with a standard deviation of four months. Both radiological and clinical union were achieved, with averages of 14 weeks and eight weeks, respectively. Impressively, no complications were observed following IM K-wire fixation, indicating positive immediate post-operative outcomes. Three participants (33.3%) had not yet undergone instrumental removal, while the remaining six (66.7%) experienced no complications following removal. In terms of post-operative pain, the majority (88.9%) reported mild or no pain during their final follow-up, while one participant (11.1%) reported moderate pain, and none presented with severe pain necessitating an emergency room visit. All participants achieved a full range of motion for their elbow, falling within the range of 10-150 degrees, which is a positive indicator of post-operative recovery. Additionally, post-operative blood transfusion was required for only one participant (11.1%) due to femur closed reduction and internal fixation (CRIF), while the majority (88.9%) did not require this intervention.

**Table 4:** Post-operative factors following IM K-wire fixation of humeral shaft fractures. n: number; SD, standard deviation; IM, intramedullary; K-wire, Kirschner wire; ER, emergency room

		n (%)
First post-operative visit (days) (mean ± SD)		20 ± 17
Time until instrumentation removal (months) (mean ± SD)		13 ± 4
Time to radiological union (weeks) (mean ± SD)		14 ± 6
Time to clinical union (weeks) (mean ± SD)		8 ± 5
Rate of radiological union		9 (100.0%)
Complications following IM K-wire fixation	None	9 (100.0%)
Rate of instrumentation removal	Removal not done yet	3 (33.3%)
	Removal done	6 (66.7%)
Post-operative pain	Mild (reported no pain in the last follow-up)	8 (88.9%)
	Moderate (still reporting pain in the final follow-up)	1 (11.1%)
	Severe (presented to ER due to pain)	0 (0.0%)
Post-operative elbow range of motion (ROM)	Full (10-150)	9 (100.0%)
Post-operative blood transfusion	No	8 (88.9%)
	Yes	1 (11.1%)

## Discussion

Fractures of the humeral shaft have a significant impact on an individual's ability to function, as well as their social and psychological well-being. The primary objective of the management team is to achieve a full recovery of functional ability within the shortest feasible timeframe. The management of humeral shaft fractures necessitates prompt and thorough restoration, ensuring unrestricted functionality to facilitate the patient's recovery of regular activities without facing financial or social difficulties. Intramedullary K-wires may be more effective than conservative therapy and immobilization methods in treating humeral shaft fractures because they allow faster rehabilitation and range of motion at the elbow. Khan et al. (2007) found that the use of several intramedullary Kirschner wires had positive clinical and functional outcomes [5]. Similarly, Qidwai's results are consistent with our findings [6].

To investigate this, we conducted a cross-sectional study involving 17 patients who met the specified inclusion criteria and did not meet any of the exclusion criteria. Eight patients were excluded due to incomplete data, leaving us with a final analysis of nine patients. The average patients' ages were 34 years with a standard deviation of 20 years. The reason this age range has been selected is because of the increased likelihood of humeral fractures associated with increased levels of physical activity. However, reports of patients' typical ages as high as 52-72 years old have been found in various studies [8,9]. Nevertheless, despite the occasional variations, the occurrence of humeral fractures tends to be more prevalent among those with a greater degree of physical activity, as seen by the age distribution of patients in the present study.

In terms of gender-based evaluation, it was found in our study that the majority of patients included in the study were male (88.9%), while the remaining patients were female (11.1%). This data supports our previous hypothesis on the correlation between the occurrence of humeral fractures and an individual's degree of physical activity. In contrast to this, Western studies indicate a higher incidence of humeral shaft fractures in females in comparison to males. The authors of these studies explain that this is due to a decrease in bone mineral density among females as they age [10]. The etiology of humeral shaft fractures varies between our setting and Western settings. In Indian studies, violence and road traffic accidents are prominent causes of humeral shaft fractures, but in Western contexts, the etiology tends to vary. Indian studies have often highlighted a prevailing male dominance in the context of road traffic accidents, which has been identified as the primary cause [11,12]. In our study, it was shown that motor vehicle accidents accounted for the highest proportion of cases (66.7%).

In the present study, middle fractures accounted for 55.6% of all fractures, with proximal fractures making up 33.3% and distal fractures making up just 11.1%. Based on a study conducted by Carroll et al. (2012), 64% of fractures were middle third, 30% were proximal third, and 6% were distal third [8]. The majority of fractures in this study were transverse or oblique (77.8%), with spiral fractures being less common (22.2%). These findings can be compared with a prospective study, which typically describes transverse or oblique with butterfly fractures as the most common types of humeral shaft fractures [13]. The location, nature, and degree of the impact that caused the fracture determine the fracture type. Injuries to the bone, such as those that produce oblique with butterfly and oblique fractures, are usually the result of a fall accident or other traumas. High-impact trauma is the most common cause of comminuted fractures, whereas bending forces are often responsible for transverse fractures [14].

The union rate in our study is 100%. This union rate shows an excellent outcome for this management technique and fixation method. The rate of non-union after nonoperative treatment can be as high as 14%-23%, and up to 29% of patients eventually undergo open reduction and internal fixation (ORIF) after nonoperative treatment [15]. Large retrospective series evaluating surgical management demonstrate union rates between 84% and 97% [3]. Thus, the union rate in our study is comparable and possibly better than that of nonoperative or plate and screw management.

The mean duration of the intra-operative procedure was 125 minutes, with a relatively small standard deviation of 23 minutes. This consistency in the duration of surgery suggests that the surgical team was efficient in conducting the procedures. This falls within the range of surgical times reported in the literature for humeral fracture fixation [16]. The average blood loss of 78 mL is noteworthy, although the standard deviation of 51 mL indicates variability among patients. Minimizing blood loss during surgery is essential, as excessive blood loss can lead to complications and prolonged recovery [17]. The absence of any blood transfusions required during the intra-operative period reflects the success of the surgical procedures in maintaining hemostasis.

The complication rate in our study was 0%. The complications studied were the failure of fixation, prominent hardware post-operatively, non-union, infection, and radial nerve palsy post-operatively. None of the patients in our case series experienced any of these complications. With plate and screw fixation, the risk of infection can reach up to 2% [18]. The reported rates of post-operative radial nerve palsy range from 3% to 7% [19].

The first post-operative visit occurred at a mean of 20 days, with a standard deviation of 17 days. This variability in the timing of the first post-operative visit could be attributed to various factors, including the individual healing process, post-operative care, and the duration of hospital stay due to other injuries or medical comorbidities. Instrumentation removal took an average of 13 months, with a standard deviation of four months. The timing of instrumentation removal is an important consideration in the management of humeral shaft fractures, suggesting that the IM K-wire fixation method was effective in promoting fracture healing. The early removal of an instrument may result in complications, while delayed removal may impede patient recovery. These findings are consistent with existing literature on the timing of removal, which suggests that early removal is not recommended [20].

In the present study, we found that a majority of patients (88.9%) reported no pain on follow-up. There were no cases of extreme pain intensity in the current study because pathological fractures were not included in the sample. Several studies have been conducted, which have shown that the movement or relocation of K-wires leads to a painful experience [9,21]. Nevertheless, the level of pain experienced by participants in our study did not suggest the occurrence of such an event. Achieving a full range of motion for the elbow in all participants is another positive outcome, as it is essential for functional recovery. These findings are in line with the prospective study, which found excellent elbow movements in 93% of patients [13].

Non-surgical management of humeral shaft fractures may be complicated by non-union and malunion [16], while surgical management with plates and screws may increase the probability of complications such as iatrogenic radial nerve palsy and infection [11]. Our study showed that the intramedullary K-wire fixation of humeral shaft fractures can have very high clinical union rates along with little to no complications. Thus, intramedullary K-wire fixation for humeral shaft fractures may show benefits over both conservative management and plate and screw fixation. The treatment method is also ideal for poor and impoverished countries because of its inexpensive cost, wide availability of K-wires (in comparison to titanium nails or plates and screws), and short hospital stay. We recommend larger and prospective studies to confirm our findings.

Limitations

The small sample size and the lack of diversity, as the majority of participants were male, may limit the ability to draw broader conclusions. The study mainly focuses on immediate post-operative outcomes and does not investigate long-term functional outcomes or quality of life. Furthermore, the study being a retrospective cross-sectional study does impose limitations on both the quantity and quality of the available data. We strongly recommend conducting a prospective study with a larger sample size to further validate and strengthen our findings.

## Conclusions

The intramedullary K-wiring technique for humeral fractures is a surgical procedure that involves the insertion of K-wires into the medullary canal of the humerus. The K-wires are then used to stabilize the bone and promote healing. This technique is quick and can prove to be highly successful. One of the key benefits of this technique is that it provides dynamic fixation without sacrificing stability. This means that the bone is stabilized but still has the ability to move and adapt to the patient's movements. This helps to promote proper healing and reduce the risk of complications such as non-union or malunion. Our study found that all patients who underwent intramedullary K-wire fixation achieved completely healed fractures and full elbow range of motion and experienced only mild to moderate pain post surgery. Furthermore, no complications such as infection or radial nerve palsy were observed. Overall, this study shows that the intramedullary K-wiring technique for humeral fractures can be a safe, effective, and affordable treatment option that can provide patients with excellent outcomes.
